# Nurses' Food, Work, and Hours of Recreation

**Published:** 1890-11-29

**Authors:** Henry C. Burdett

**Affiliations:** Deputy Chairman of "The Hospitals Association," &c., &c., &c.


### Nurses' Food, Work, and Hours of Recreation.

By Henry C. Burdett, Deputy Chairman of "The Hospitals Association," &c., &c., &c.

I am old enough to remember hospitals and nurses as they were when Miss Nightingale first commenced her crusade of charity and reform. Looking back only a quarter of a century, however, those of us who know the state of affairs then and now, may thank God and take courage.

### The Growth of Nursing.

In 1868 it was asserted that our attempt to introduce young and intelligent women as probationer nurses into a large provincial hospital was foredoomed to failure on moral, social, and physical grounds. For years the proportion of applicants wko failed after a preliminary trial was enormous, and it is surprisingly large still. At the outset, from some records I kept at the time, I found that out of one hundred women who applied to be admitted as probationers, only four became trained and certificated nurses. Mrs. Wardroper used to say that at the Nightingale School from thirty to forty out of every hundred probationers would, from one cause or another, fall out of the ranks during the period of training. It is desirable to remember these facts, as they illustrate how many who desire to leave home from one cause or another—very many young women regard nursing from a distance as an easy and pleasant pastime—are in practice utterly unsuited for the calling of a nurse. Few callings are more honourable, but I believe none to be so exacting and onerous.

Who is it that has made it possible for the public to have trained nurses at all? Who have raised the status of the nurses' calling, and by wise regulations and thoughtful care made nursing what it is to-day? Who are the nurses' best friends, to whom must the medical man and the head of a family look when it is essential to have a specially qualified nurse for a particular case? Surely to the Nurse Training Schools. Hence the existence and efficiency of the Nurse Training Schools are essential to the safety and welfare of the nurses and also of the public. The matron of a training school knows exactly where a particular pupil excels and where she is wanting. And as the duties of a nurse consist in tending the sick, as opposed to those of the doctor, which involve the treatment and the prescribing of medicine, so it is essential to the comfort, nay, sometimes to the very recovery of a patient, that the nurse selected should possess the qualities and capacity for tending the case in the best and special way the doctor requires. The nurse is sure of better and juster treatment at the hands of her *Alma Mater* than from others, because her school authorities know all about her, and it is to their interest, as well as to her own, that she should always give satisfaction, and so achieve the maximum of success. Neither the medical profession, the nurses, nor the public can do without the Nurse Training School, and it behoves us to unite these four interests in every possible way. A very great responsibility, therefore, rests upon the Nurse Training School authorities, and I will now proceed to consider how far their action has justified Miss Nightingale in her verdict that the recent charges are without foundation. I have divided my subject into three heads; and purpose to consider each in turn. To enable me to do justice to the subject, I placed myself in communication with all the great hospitals (metropolitan and provincial), and some of the representative Poor Law Infirmaries, and I am deeply indebted to the matrons and secretaries who have so kindly supplied me with the facts I am about to deal with.

### Nurses' Food.

The statement has been made, and widely circulated, that the food supplied to nurses, and especially to night nurses, is insufficient and unsuitable. The grounds for this assertion are, that on one night in a particular hospital the night nurses on duty had sardines and marmalade for breakfast before going on duty, and pickled mackerel with bread and butter to sustain them during the night. It is interesting in this connection to point out that when Miss Twining read a paper before this Association in May, 1885, she suggested a reduction in the amount of meat, and the substitution of farinaceous food and soup in lieu of the best joints, poultry, &c. Here, therefore, is an amazing conflict of opinion, with the notable circumstance that the trained and experienced authority, Miss Twining, demands less meat, whilst the new-born enthusiasm of the amateur and inexperienced critic declares such food to be insufficient and unsuitable, and so cries out for meat, more meat.

The regard which is shown for the varied tastes of the nurses seems proved by such notes as the following: "Meat for nurses in diphtheria wards," "Variety studied as much as possible," "Have very few routine dishes, and vary puddings as much as possible." Even the ancient tradition concerning Friday is remembered, for I find the note from one hospital runs: "Friday, hot fish and cold meat;" and again, that from another: Fish one end of table on Friday, and meat at the other." The preponderance of fish on Fridays in a hospital like the London, which numbers many Roman Catholics on its staff, is solely due to consideration for the nurses. I have only one other remark to make on this head. It is proverbial that a bountiful Creator sends us food, and someone else, who shall be nameless, sends us cooks. Now, although I am convinced, from personal observation, that the cooking has everywhere vastly improved, owing, in the case of the larger hospitals, to the establishment of a separate kitchen and staff for the nurses, still, no doubt, the best of cooks will everywhere find a hearty welcome. An obliging correspondent, an able hospital secretary, suggests, therefore, that, as it is difficult to get a good cook, it might be well if some of the thousands of applicants who aspire to be nurses would turn their attention to cooking, and then everybody ought to be pleased and thankful.

Speaking generally, I should venture to state that at the larger hospitals throughout the country the food arrangements for nurses are decidedly good and sufficient. No doubt individual governors might with advantage call at the hospitals they may support, any day about a quarter to one, the hour at which the nurses' dinners are served. They would, I know, be welcomed by the matron, who would show them into the nurses' dining-room, and so they would be able to judge for themselves. Hospital committees would be well advised to take care to remember the importance to be attached to a knowledge of housekeeping on the part of the matron when making such appointments. It has not been possible for me to go into the food supply of nurses at the smaller hospitals, some of which it is declared have not yet displayed sufficient interest in this important question. Be this as it may, I hope one result of this paper may be that every hospital committee and matron, however small the institution, will look into this matter with a view to remedying any defects, should such be found to exist.

### Hours of Work.

In considering this point we must remember that nurses are not nearly so hard worked nowadays as they were formerly. There must now be at least two, and sometimes even three nurses on the permanent staff for every one formerly employed. Then the work used to be far harder and rougher in every way. In order to understand the labour demanded of a nurse, it is necessary to remember that there are hospitals and hospitals. In non-clinical hospitals—*i.e.*, hospitals not connected with medical schools—the work is usually much lighter than in others. Again, in clinical hospitals the students do much of the work which devolves upon the nurses at the smaller institutions. I am by no means certain that some non-clinical hospitals do not turn out the best nurses in consequence. Then, in the provincial hospitals it is usual for the honorary medical staff to visit the wards in the early part of the day, the consequence being that in the afternoon the work is light, and a nurse, as a result, has much more time to herself. In the large London hospitals, on the contrary, the honorary medical staff visit the wards in the afternoon, a practice which greatly increases the strain on the nurses employed. These and many other considerations must be borne in mind, as it is impossible to deal with this question as affecting all hospitals alike, or to compare any two hospitals without a full knowledge of the special features of each. I should have been in a difficulty on this point, as I have purposely avoided mentioning any particular hospital, had it not been for the fact that Miss Isla Stewart has written an excellent article in *Murray's Magazine*, for August—which everyone interested in these questions ought to read—in which she gives full particulars concerning the hours of work at St. Bartholomew's Hospital.

Miss Stewart tells us that probationers at Bart.'s during their three years' training are off duty each month, during the first week on three days only, viz., twice for two and three-quarter hours, and once for three hours. During the second week once for two and three-quarter hours, and once for six and three-quarter hours. In the third week twice for two hours and three-quarters, and once for three hours, and in the fourth week once for two and three-quarter hours, and once for thirteen and three-quarter hours. They also are allowed three hours off duty on Sundays.

It will thus be seen that at St. Bartholomew's Hospital on three days in each week, and sometimes for four days in each week, the nurses are kept on duty for fourteen hours in each day, with no intermission, except at meal times, and there can be no question that Miss Stewart is right in stating by implication that the nurses have reason to grumble at the shortness of their off duty time.

Every hospital nurse ought to have two hours off duty every day at the very least, and in addition to this she should have one day and night off (*i.e.*, a clear twenty-four hours) per month. In the case of probationers, I think it would be wise to make arrangements to allow them to have three hours per day off duty, in addition to one day and night off per month. It is satisfactory to note that very many hospitals have recognised this fact, and that it is the practice to allow nurses at least two hours per diem off duty, in addition to meal times. I am of opinion that a fair day's work for a nurse is ten hours, the working day to extend from 7 a.m. to 9 p.m.

One word as to the night nurses. It is the almost universal opinion of hospital matrons at the present day that night duty should be undertaken for a period of three months, and no longer, when day duty should be resumed. Thus, in some hospitals a probationer is put on night duty for three months, and then on day duty for a similar period in the same ward. She is then moved to another ward, where she serves three months on night duty, and three months on day duty as before.

### Hours of Relaxation.

I have already stated that every nurse should be allowed at least two hours per diem for relaxation, in addition to one day off duty per month. In addition to the two hours per diem, some hospitals, the London amongst others, have the excellent arrangement of giving the sisters power to grant a nurse four hours off duty once a week, as circumstances will permit: the matron has the power to grant late passes to those who wish to go to a concert, or perhaps dine with friends. Every nurse should be allowed three weeks' holiday in the year, which should be taken in periods of not less than one week at a time. Sisters should have at least thirty-one days' leave, to be taken in the same way and under similar conditions.

The question arises, how can nurses spend their leave, and what can they do with themselves in the heart of a great city? I find that they are, not unnaturally, so tired that most of them do not care to walk. The top of an omnibus or tramcar, and the deck of a steamboat, in fine weather, are therefore very favourite methods of taking the air. When a nurse has four hours, she likes to get away to the West-end to see the shops, the picture-galleries, or to go to an afternoon concert or theatre. I am inclined to think, however, that much good might be done in an inexpensive way by many wealthy people if they were to give a thought to the busy and exacting life that nurses lead in our hospitals. In summer, at very little expense, one or more of the wealthy governors might arrange to give the nurses attached to the hospital they favour, an occasional picnic to Kew Gardens, to their own country seat, or to some other locality where fresh air and country scenes can be provided. Again, an occasional theatre does the nurse a great deal of good, and I think I may with confidence bring this fact to the notice of the managers. The managers of some of the principal theatres already send tickets to the matrons for the nurses from time to time. Many people have boxes at the Albert Hall, at Drury Lane, and elsewhere, which they cannot always occupy, and which might be filled by hospital nurses and sisters, if the owners would send their pass in turn to the matrons of the various hospitals. It has been suggested that ladies and gentlemen might send their carriages to the hospitals by arrangement with the matron, and so give nurses an airing.

When a nurse goes for a holiday it is essential for her health that she should escape from what is vulgarly known as "the shop." She does not want to meet other nurses in some central home, where the talk will be eternally about hospitals and cases, and the many details of the daily life and work she leads in the hospital. She wants rest, she wants change of scene, of ideas, of companions, and of surroundings. Without these her holiday is relatively unsuccessful, and her health is not re-established to anything like the extent it would have been had she avoided institution life, and gone elsewhere. For these reasons, I believe that the scheme propounded by Miss Philippa K. Hicks, of the Great Ormond Street Hospital for Children, which has proved such a great success, is one worthy of the generous support and cordial approval of all who are interested in nurses. Experience shows that when sadly needing a few weeks' rest and holiday, many nurses have neither friends nor relations whom they can visit, hence Miss Hicks started the idea of getting ladies in the country to invite one or more nurses during the year to stay in their families. By this means a more complete time of rest, relaxation, and change is obtained than by a far longer stay in some institution.

The plan has been tried for some years, and has proved so great a boon to the nurses in many of our large City hospitals, that the time has arrived when an endeavour should be made to secure its extension by getting more ladies to offer a fortnight's change to those who need it. The ladies who have so far co-operated, state that in large houses such an addition to the household makes no difference, whilst others, who have but small means and large hearts, full of charity and love towards those whose lives are devoted to the sick and suffering, maintain that the admission of the nurse into their household for a short time is no inconvenience. Miss Hicks is a practical woman, who carefully arranges the details and sends her nurses to houses where the circumstances of hostess and guest will best assimilate. The scheme deserves the utmost measure of support and sympathy, because it enables nurses to get quite away from the sad routine of sick nursing into fresh scenes and new surroundings, which is the most invigorating stimulant to renewed energy for work.

General Summary.

I will now summarise the opinions of the ladies who have charge of the principal hospitals in this country.

It is considered that the remedy for long hours will be found in the proper arrangement of the work of the nursing staff. It is generally held that for each set of wards containing thirty beds there should be provided one sister, one staff nurse, and three probationers.

It is generally allowed that ten hours on duty in the wards is not too long a time, and that to reduce the hours below this point would have very serious disadvantages, and not be calculated to promote the best interests of either the nurses, the patients, or the hospitals. After all, it is essential to good nursing that each sister and the charge-nurse should take a personal interest in their cases, and to secure this they must have practical charge of them from first to last, for which purpose it is necessary that they should be on duty for at least ten hours in each day.

On the question of food, where there is a nursing institution attached to the hospital and a nurses' home, it seems desirable that there should be a separate kitchen and *cuisine* for the nurses. Although a printed diet table setting forth the meal to be provided for the nurses each day for the period of a month has advantages, it is generally held that as as much variety as possible should be aimed at, the better plan is to keep a record of the meals supplied each day, without a fixed diet table.

The best arrangement of meals of night nurses appears to be: Breakfast from eight to half-past eight p.m.; two meals during the night, and dinner on coming off duty at nine to half-past nine a.m. In some hospitals the night nurses have milk, or some light refreshment, before going to bed. The meals during the night are served in the ward kitchens, breakfast and dinner being served in the nurses' dining-hall.

Although some matrons hold that it is better for a nurse to be permanently engaged on night duty, the general opinion seems to be that it is best for the health of the nurses and for the efficiency of the nursing that they should alternate day and night duty, changing over every three months.

Finally, there seems to be a general consensus of opinion that the matron should either preside during the nurses dinner hour, or that she should, at any rate, frequently inspect the meals served in the nurses' dining-hall.

